# Aberration correction for improving the image quality in STED microscopy using the genetic algorithm

**DOI:** 10.1515/nanoph-2018-0133

**Published:** 2018-11-13

**Authors:** Luwei Wang, Wei Yan, Runze Li, Xiaoyu Weng, Jia Zhang, Zhigang Yang, Liwei Liu, Tong Ye, Junle Qu

**Affiliations:** Key Laboratory of Optoelectronic Devices and Systems of Ministry of Education and Guangdong Province, College of Optoelectronic Engineering, Shenzhen University, Shenzhen 518060, P.R. China.; Key Laboratory of Optoelectronic Devices and Systems of Ministry of Education and Guangdong Province, College of Optoelectronic Engineering, Shenzhen University, Shenzhen 518060, P.R. China.; State Key Laboratory of Transient Optics and Photonics, Xi’an Institute of Optics and Precision Mechanics, Chinese Academy of Sciences, Xi’an 710119, P.R. China; Key Laboratory of Optoelectronic Devices and Systems of Ministry of Education and Guangdong Province, College of Optoelectronic Engineering, Shenzhen University, Shenzhen 518060, P.R. China.; Key Laboratory of Optoelectronic Devices and Systems of Ministry of Education and Guangdong Province, College of Optoelectronic Engineering, Shenzhen University, Shenzhen 518060, P.R. China.; Key Laboratory of Optoelectronic Devices and Systems of Ministry of Education and Guangdong Province, College of Optoelectronic Engineering, Shenzhen University, Shenzhen 518060, P.R. China.; Key Laboratory of Optoelectronic Devices and Systems of Ministry of Education and Guangdong Province, College of Optoelectronic Engineering, Shenzhen University, Shenzhen 518060, P.R. China.; Department of Bioengineering and the COMSET, Clemson University, Clemson, South Carolina 29634, USA; Key Laboratory of Optoelectronic Devices and Systems of Ministry of Education and Guangdong Province, College of Optoelectronic Engineering, Shenzhen University, Shenzhen 518060, P.R. China.

**Keywords:** aberration, adaptive optics, genetic algorithm, super-resolution imaging

## Abstract

With a purely optical modulation of fluorescent behaviors, stimulated emission depletion (STED) microscopy allows for far-field imaging with a diffraction-unlimited resolution in theory. The performance of STED microscopy is affected by many factors, of which aberrations induced by the optical system and biological samples can distort the wave front of the depletion beam at the focal plane to greatly deteriorate the spatial resolution and the image contrast. Therefore, aberration correction is imperative for STED imaging, especially for imaging thick specimens. Here, we present a wave front compensation approach based on the genetic algorithm (GA) to restore the distorted laser wave front for improving the quality of STED images. After performing aberration correction on two types of zebrafish samples, the signal intensity and the imaging resolution of STED images were both improved, where the thicknesses were 24 μm and 100 μm in the zebrafish retina sample and the zebrafish embryo sample, respectively. The results showed that the GA-based wave front compensation approach has the capability of correction for both system-induced and sample-induced aberrations. The elimination of aberrations can prompt STED imaging in deep tissues; therefore, STED microscopy can be expected to play an increasingly important role in super-resolution imaging related to the scientific research in biological fields.

## 1 Introduction

Due to the nature of light diffraction, the lateral resolution of a light microscope is limited to approximately half the wavelength, which is typically equivalent to 200–350 nm in the visible light region [1, 2]. Fortunately, the advent of fluorescence-based super-resolution microscopy (SRM) techniques makes it possible for us to observe the details of nano-scale structures such as viruses and subcellular structures and may eventually transform our view of cellular and molecular biology [3–5]. As one of the powerful SRM techniques, stimulated emission depletion (STED) microscopy has made significant achievements in biomedicine and in many relevant applications [6–9]. Compared with other SRM techniques, including stochastic optical reconstruction microscopy [10, 11], photoactivated localization microscopy [12, 13], and structured illumination microscopy [14, 15], STED microscopy has the advantages of relatively simple sample preparation procedures and a fast imaging speed with no post-reconstruction required. In STED microscopy, super-resolution imaging is achieved by superimposing a donut-shaped depletion focus on a Gaussian excitation focus. The donut-shaped laser beam (called the depletion beam) at the red-shifted wavelength relative to the peak of the emission spectrum suppresses the fluorescence at the periphery of the excitation focus spot to effectively narrow the point spread function (PSF) below the diffraction limit.

Ideally, the center intensity of the donut-shaped depletion beam should be 0. In this case, the effective PSF is reduced infinitely with the increase in depletion intensity. However, the performance of STED microscopy critically depends on the quality of the depletion beam, especially the intensity distribution symmetry and the contrast between the central minimum intensity and the surrounding illumination. As a result, the actual resolution in STED microscopy is very susceptible to aberrations brought by the optical system and biological samples, which distorts the wave front of the depletion beam in the focal plane. The system-induced aberration is basically fixed in a certain optical system, but the sample-induced aberration is complicated and unpredictable because of the surface roughness and the inhomogeneity of the refractive index [16, 17]. Unfortunately, aberrations exist in all types of optical imaging, which defocus and diffuse the laser focusing spot. In recent years, the influences of aberrations on STED imaging have been studied [18–21]. The results indicated that aberration correction should be given priority to consider in STED imaging, especially in the situation of a high depletion power and in the deep tissue imaging.

The purpose of aberration correction is to restore the original laser wave front after propagating a certain distance. An important method for aberration correction is adaptive optics (AO), which was first applied to compensate for the distorted wave front induced by atmosphere turbulence [22, 23]. In previous reports, AO has been used in STED microscopy for aberration correction based on different approaches [24–28]. However, some of these studies only focused on correcting the aberrations induced by optical components [24, 25]. These approaches have the capability to compensate for the system-induced aberration but overlook more complicated aberrations induced by biological samples. Some other studies have achieved full aberration correction, i.e. the system-induced aberration and the sample-induced aberration in biological samples [26–28]. However, the imaging depth is still small (a few micrometers to over 20 μm). Meanwhile, the use of multiple adaptive elements in these studies, usually two spatial light modulators (SLMs) or the combination of a deformable mirror and an SLM, increases the complexity and the expenditure of the system.

In this work, we present a wave front compensation approach based on the genetic algorithm (GA) to find the corrected phase. In the experiments, the modulation of the wave front phase and the conversion of the depletion beam from Gaussian to donut-shaped were controlled by only one SLM, which allowed for aberration correction without any modification to the original STED system. By using the back-scattering signal of gold nanoparticles as feedbacks in the GA optimization, the distorted wave front of the depletion beam can be rapidly restored. Previous studies on wave front shaping have shown that the GA is an efficient and robust method that can optimize many phase segments in parallel and maintain a high stability in the noisy environment [29]. We have shown that the signal intensity and the imaging resolution of STED images can be improved after aberration correction.

## 2 Methods

**Figure 1:**
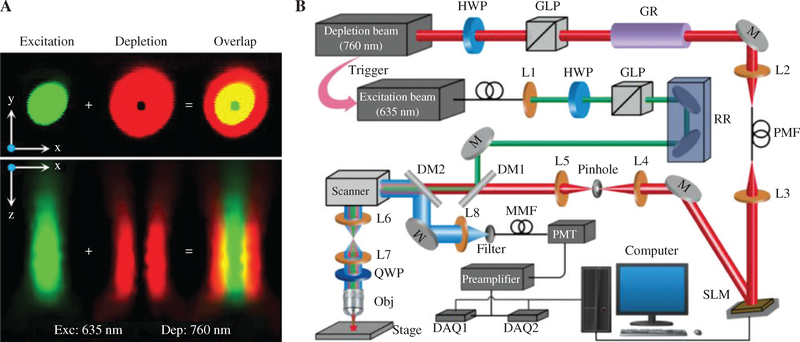
Principle and experiment system of STED microscopy. (A) Focal spots of the excitation beam and the depletion beam, and their overlap in both XY plane and XZ plane. (B) Schematic of the STED imaging system. L, lens; HWP, half wave plate; GLP, Glan-laser polarizer; GR, glass rod; RR, retro reflector; M, mirror; DM, dichroic mirrors; Obj, objective lens; QWP, quarter wave plate; SMF, single mode fiber; PMF, polarization maintaining fiber; MMF, multimode fiber; DAQ, data acquisition device.

In a STED microscope, a donut-shaped depletion beam is delivered to a home-built confocal laser scanning microscopy system to quench the fluorescence from the peripheral area of the emission spot through stimulated emission. As shown in Figure 1A, the focal spots of the excitation beam and the depletion beam overlapped in space to compress the effective PSF. Increasing the depletion laser power can decrease the emission area of fluorescence for resolution improvement. However, it could cause a non-negligible intensity in the center of the depletion beam due to aberrations, which has negative effects on the image quality such as imaging resolution and signal intensity. The image quality is worse after combining the complexity within a thick sample. Therefore, a depletion beam with a high quality is crucial for STED imaging. To carry out aberration correction for depletion beam, only one SLM with no additional adaptive elements was used in this work. The schematic of the experiment system is shown in Figure 1B, which is composed of three light paths: excitation, depletion, and detection.

The excitation beam with the wavelength of 635 nm was provided by a picosecond diode laser head (LDH-D-C-635, PicoQuant, Berlin, Germany), and a widely tunable, mode-locked Ti:sapphire laser (Chameleon Ultra II, Coherent, Santa Clara, CA, USA) with a temporal width of 140 fs was tuned to 760 nm as the depletion beam at a pulse repetition rate of 80 MHz. In the excitation path, the repetition rate and laser intensity of the excitation beam were modulated by a laser drive (PDL 800-D, PicoQuant, Berlin, Germany), which was externally triggered by the depletion laser source. In order to maintain linear polarization and adjust the laser intensity in both excitation and depletion paths, a half wave plate (Thorlabs, Newton, NJ, USA) and a glan-laser polarizer (Thorlabs, Newton, NJ, USA) were used as a combination. Then, a delay line controlled by a retro reflector (Thorlabs, Newton, NJ, USA) in the excitation path adjusted the interval of the two series pulses for a better performance of STED imaging. In the depletion path, the wavelength of the depletion beam modulated by Ti:sapphire laser can be tuned from 680 nm to 1080 nm. A glass rod (Schott, Mainz, Germany) and a 100 m single-mode polarization maintaining fiber (Thorlabs, Newton, NJ, USA) temporally stretched depletion pulses to approximately 200 ps. A SLM (PLUTO-NIR-011, HOLOEYE Photonics AG, Berlin, Germany) as an important adaptive element was introduced for the generation of donut-shaped depletion beam and aberration correction. In order to eliminate the influence of stray light, a blazed grating phase was always loaded on the SLM to form a series of diffraction spots. A pinhole was placed in the focus of the lens (L4) to select the first-order diffraction spot. The excitation beam and the depletion beam encountered and overlapped at the dichroic mirror DM1. After a pair of galvanometer scanning mirrors (6210H, Cambridge Technology Inc., Cambridge, MA, USA), a quarter wave plate (Thorlabs, Newton, NJ, USA); converted the depletion beam to right-hand circular polarization for better imaging resolution. Afterwards, the sample was fixed in a 3D stage (MPC-385 Series, Sutter Instrument Company, Novato, CA, USA) with full travel of 25 mm and a minimal step size of 62.5 nm in each axis. An oil-immersion objective (HCX PL APO, 100 ×/1.40–0.70 OIL, Leica, Wetzlar, Germany) focused two laser beams on the sample and collected the fluorescence signal. The detection path allowed the transmission of fluorescence signal to a photomultiplier tube (PMT; H7422–40, Hamamatsu Photonics, Japan). A preamplifier was placed before the computer to amplify the signal, and the output signal was transmitted to two data acquisition devices (PCI-6110/USB-6351, National Instruments, Austin, TX, USA) for imaging and aberration correction, respectively. An open-source program, Scan-Image (HHMI/Janelia Farm, Ashburn, VA, USA) developed in MATLAB (Mathworks, Natick, MA, USA), was used to control the scanning mirrors and acquire images.

**Figure 2:**
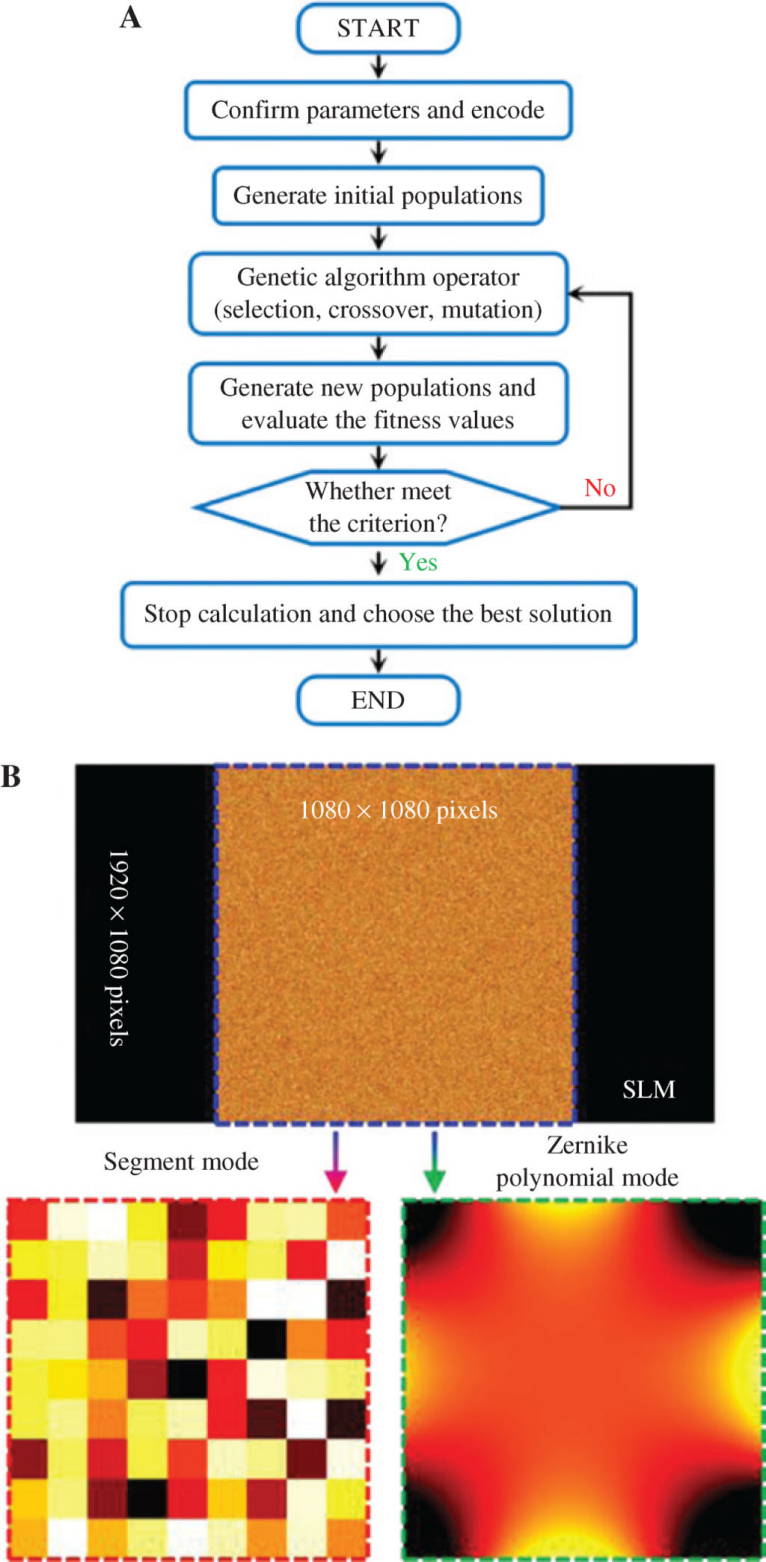
Principle of genetic algorithm. (A) The flowchart of genetic algorithm for finding the best solution. (B) Two encoded forms on SLM in GA procedure.

Inspired by the natural species evolution, GA is a stochastic, parallel method to search the optimal solution globally. As an efficient evolutionary algorithm, it can simulate the behaviors of biological populations (e.g. selection, crossover, and mutation) [30, 31]. By encoding the genes of individuals, each individual corresponds to a fitness value. It can be treated as the criterion to compare the individual differences. After the evolution of several generations, one individual with the optimal fitness value is selected. Figure 2A shows the flowchart of the GA for finding the optimal solution. There are several basic concepts in GA: the individual (the solution of an un-optimized problem); the gene (the encoded form of individuals); the fitness value (the criterion to evaluate the difference between individuals); the population (all individuals in a fixed problem); the parent (individuals in the last generation for producing the new population in the next generation); selection, crossover, and mutation (the simulation of a biological evolution process, the main operator of the GA); and the offspring (new individuals after the genetic operation from parents).

To assess the correction effect and find the optimal corrected phase, the voltage value converted from the back-scattering signal of a gold nanoparticle was treated as the criterion for the image quality metric. An SLM mounted in the depletion path serves three different purposes: first, it loads a blazed grating phase to eliminate the influence of stray light on the image quality; second, it loads a spiral phase to generate a donut-shaped depletion beam; and last, it changes the phase level to compensate for the wave front distortion. The SLM was controlled by MATLAB software (Mathworks, Natick, MA, USA).

As shown in Figure 2B, the resolution of the liquid crystal display is 1920×1080 pixels, and only the middle area (1080×1080 pixels) was invoked to improve the update rate. In the GA procedure, pixels were encoded as genes, and 1080×1080 genes made up an individual. Then, dozens or hundreds of individuals constituted a population, where the number of individuals in a population can be determined based on the complexity of the unresolved problem. The phase level of pixels on the liquid crystal display can be changed from 0 to 255, so pixels with different phase levels represented different individuals. By using gold nanoparticles as the target, the scattering signal within a certain individual was transformed to the voltage value. Therefore, the purpose of the GA procedure is to find the optimal voltage during the simulation of the biological evolution process.

However, there are more than a million genes in an individual. It can cause a slow convergence rate and a tendency for prematurity [29]. In order to resolve these problems, the control region on the SLM was modeled in the segment mode and/or the Zernike polynomial mode [25, 26]. After many experiments, the results showed that either of the two modes alone can be used in correcting the system-induced aberration, and the combination of the two modes is more suitable for full aberration correction in biological samples. In the segment mode, the control region (1080×1080 pixels) was divided into n^2^×9 segments (n is a positive integer). Each segment has the same number of pixels with the same phase level (e.g. 9×9 segments in Figure 2B). In the mixed mode, the phase level is described by the combination of the segment phase and the Zernike polynomial phase. In the experiments, aberration correction was performed on the Gaussian depletion beam. By combining the corrected phase with the spiral phase, the corrected donut-shaped depletion beam can be obtained. A blazed grating phase with a fixed period was always loaded on the SLM. Therefore, the final phase is the combination of the blazed grating phase, spiral phase, and correction phase. The wavelength of the depletion beam was tuned to 760 nm, and the scattering light from gold nanoparticles was collected as the feedback for the GA procedure.

## 3 Results and discussion

### 3.1 Correcting the system-induced aberration with GA

To verify the capability of the GA to correct the system-induced aberration in the depletion beam path, the first step is to make a comparison of the back-scattering intensity of a gold nanoparticle before and after the aberration correction. For this purpose, a sample was prepared by successively attaching gold nanoparticles (150 nm in diameter, Fisher Scientific, Vantaa, Finland) and fluorescent microspheres (100 nm in diameter; TetraSpeck™ microspheres, 0.1 μm, blue/green/orange/dark red, Invitrogen, Carlsbad, CA, USA) to a coverslip and mounting the coverslip to a microscope slide with a mounting medium (97% 2,2′-thiodiethanol, TDE, Fisher Scientific, Vantaa, Finland).

**Figure 3:**
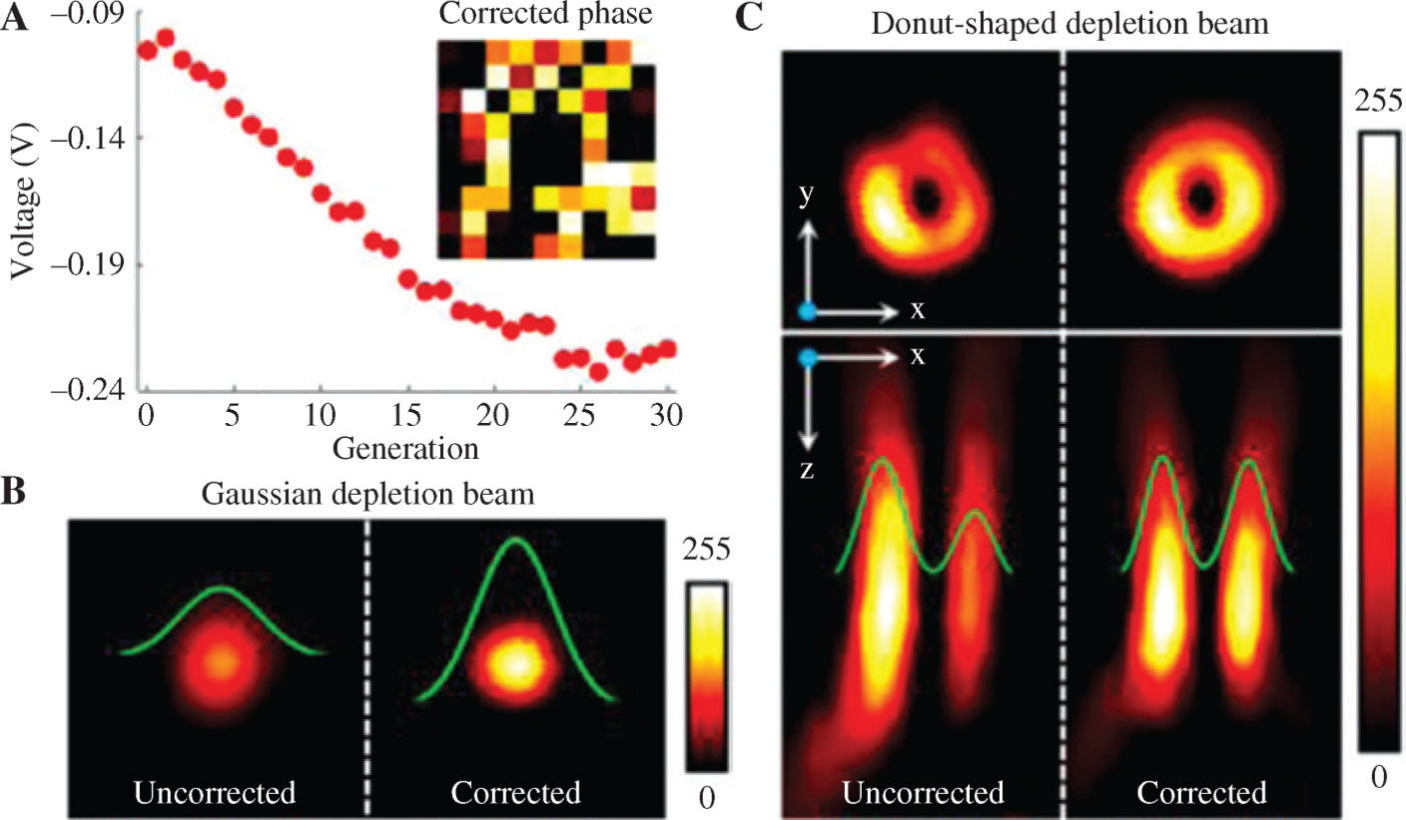
The correction of system-induced aberration by genetic algorithm. (A) The voltage change during the correction and the final corrected phase. (B, C) The comparison of scattering images of a gold nanoparticle between before and after aberration correction illuminated by Gaussian and donut-shaped depletion beam, respectively. Notes: green profiles in (B) and (C) are respective fit curves of normalized intensity.

Figure 3A shows the voltage change during the correction (transformed from the back-scattering light of a gold nanoparticle) and the corrected phase. The voltage from PMT is negative, so it decreased with the increasing iterations (the absolute value increased). After dozens of iterations, the voltage started to reach a stable state. It is worth noting that the absolute value of the voltage became smaller during the first few iterations. The reason is that the phase levels in the first generation are randomly generated, resulting in a process for pixels to find the appropriate phase level. The voltage change revealed that aberrations exist in the depletion path, and the corrected phase indicated that the wave front distortion is mainly from the spherical aberration or defocus. By imaging the same gold nanoparticle with a Gaussian depletion beam, we can see that the scattering intensity increased because the focusability of the depletion beam improved after compensating for the distorted wave front (Figure 3B). The corrected donut-shaped depletion beam can be obtained by combining the 0 ~ 2π spiral phase and the corrected phase on the SLM. As shown in Figure 3C, the intensity distribution symmetry of the donut-shaped depletion beam improved in both the XY plane and the XZ plane after performing the correction of the system-induced aberration.

**Figure 4:**
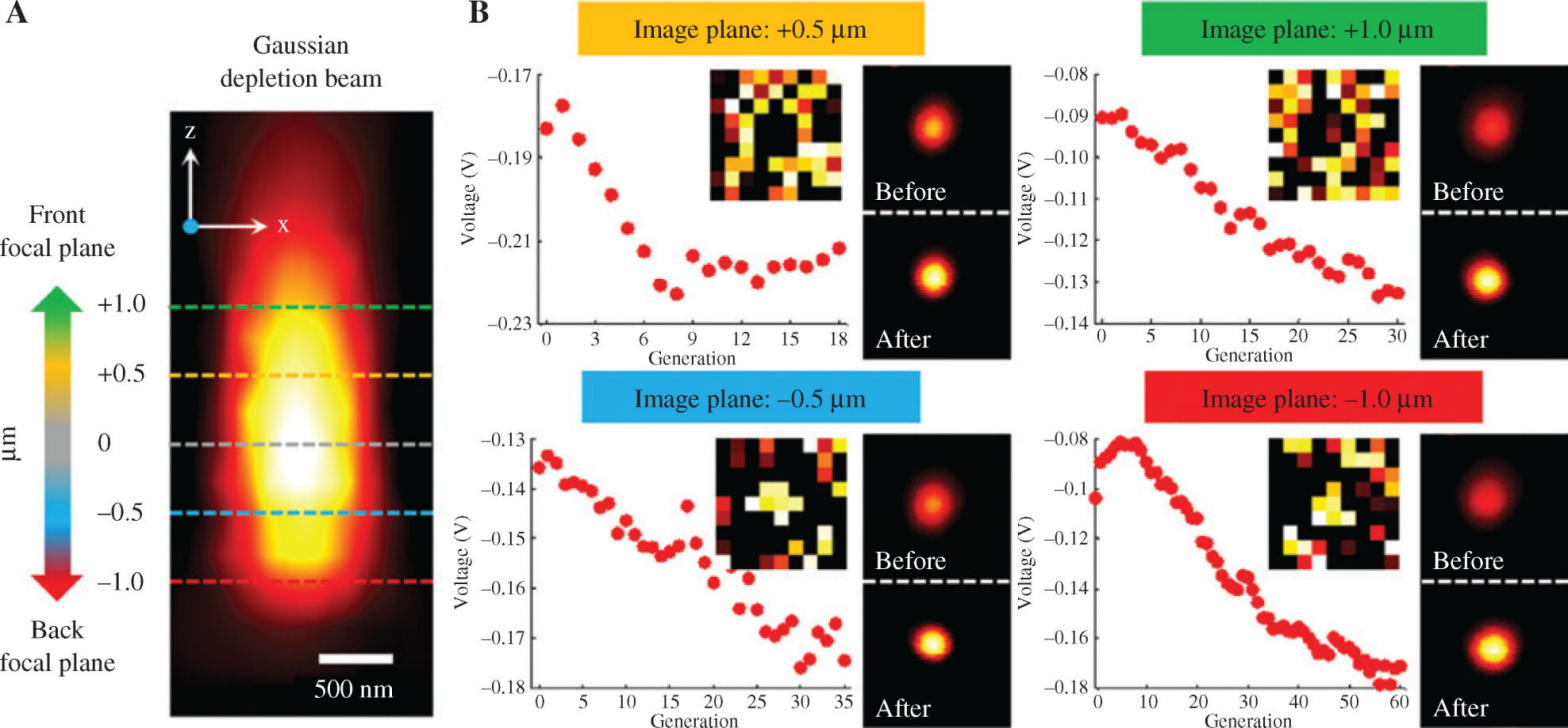
The verification of GA in correcting the artificial system aberration. (A) Scattering image of a nanoparticle illuminated by Gaussian depletion beam in XZ plane. (B) Aberration corrections at different defocusing planes (The voltage changes and the corrected phases, as well as the comparisons of scattering images of a gold nanoparticle between before and after aberration correction illuminated by Gaussian depletion beam).

The correction of more complicated aberrations can better verify the capability of the GA (e.g. the artificial system aberration by making the imaging plane out of the focal plane). Therefore, the gold nanoparticle was imaged at ±0.5 μm and ±1.0 μm away from the focal plane (Figure 4A). The absolute values of the voltage in these four cases were increased, presenting a highly focused focal spot by imaging the same nanoparticle (Figure 4B). The farther away the image plane is from the focal plane, the larger the number of iterations the GA procedure has to perform. In addition, the correction of defocus at the back focal plane required more iterations than that at the front focal plane. Notably, the corrected phases of the front focal plane and the back focal plane were inverse to each other.

In the experiments above, the control region on the SLM was divided into 81 segments (9×9 segments) to improve the calculation speed and the convergence performance of the GA procedure, of which each segment has the same phase level. A larger segment number should certainly provide a more accurate correction of the wave front distortion; however, it will take more iterations to find the optimal solution because of the slow convergence rate. More experiments were performed for different segment numbers, which is shown in Figures S1 and S2 of the Supporting Information. In consideration of obtaining a better result in less time, 81 segments are used to correct the system-induced aberration in the depletion path.

**Figure 5:**
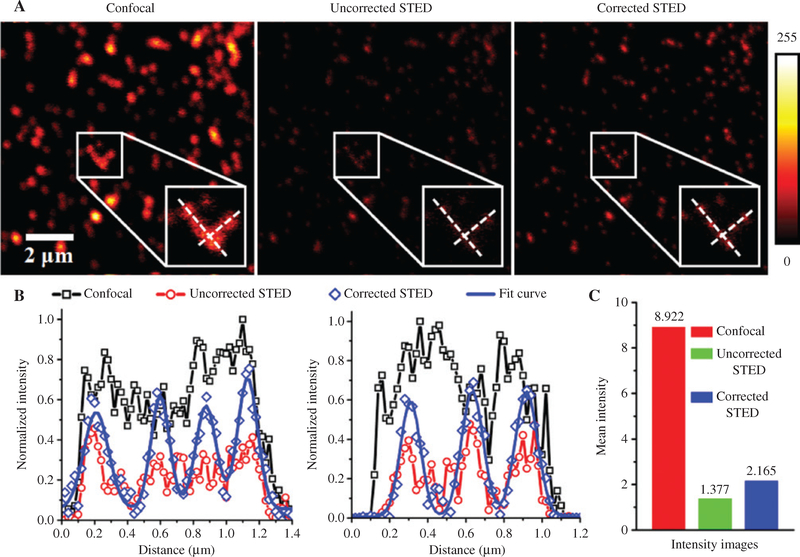
The improvement of image quality of fluorescent microspheres in STED microscopy. (A) Confocal and STED images at the depletion power of 19 mW before and after correcting system-induced aberration. (B) Normalized intensity profiles along two white dotted lines in (A), respectively. (C) The mean intensity of three fluorescence images.

After correcting the system-induced aberration, we can observe the improvement of image quality by comparing the STED images of the fluorescent microspheres before and after the aberration correction. As shown in Figure 5A, many microspheres in the confocal image are too close to be distinguished due to the diffraction limit, especially those located in the white area. Although the resolution of the uncorrected STED image by visual inspection was slightly better than that in the confocal image, the signal intensity was much lower. However, both the imaging resolution and the signal intensity in the corrected STED image had a significant improvement compared to that in the uncorrected STED image, which can be observed from the normalized intensity profiles along the two dotted lines in Figure 5B. The mean intensity of the three fluorescence images is shown in Figure 5C, and the increase in mean intensity from 1.377 to 2.165 illustrates that the correction of the system-induced aberration in the depletion path can increase the signal intensity. Therefore, the GA provides a promising strategy to correct the system-induced aberration. In the experiments, the excitation power was 45 μW at the wavelength of 635 nm, and the depletion power was 19 mW at the wavelength of 760 nm (the powers were measured at the back aperture of the objective lens).

### 3.2 Full aberration correction in biological samples

**Figure 6:**
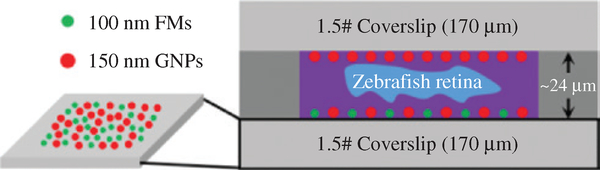
Zebrafish retina sample. Red balls are 150 nm diameter gold nanoparticles, and green balls are 100 nm diameter fluorescent microspheres.

To further prove the capability of GA in simultaneously correcting system-induced and sample-induced aberrations, i.e. full aberration correction, two types of zebrafish samples were firstly prepared, including zebrafish retina and zebrafish embryo sections. As shown in Figure 6, gold nanoparticles (150 nm) were attached to the upper 1.5# coverslip, and the mixture solution of gold nanoparticles and fluorescent microspheres (100 nm) was attached to the lower one. The zebrafish retina was sandwiched between the two coverslips and mounted in TDE. The distance of ~24 μm between the two coverslips represents the thickness of the retina, which is confirmed by the distance between two layers of gold nanoparticles in the optical axis (Z axis). The preparation of the zebrafish embryo sample used steps similar to that of the zebrafish retina sample, and the samples were prepared with different section thicknesses (the zebrafish embryos in the experiments were in the phylotypic stage).

When the laser beam passed through the retina sample, the intensity of the laser focus gradually decreased with the depth because of light scattering and absorption. The situation is worse for the depletion beam than for the excitation beam because of the donut-shaped wave front. Since the sample-induced aberration is more complicated and unpredictable, the control region on the SLM was modeled in a mixed mode of the segment mode and the Zernike polynomial mode. After performing the full aberration correction on the depletion beam in the zebrafish retina sample, the scattering intensity of the bottom gold nanoparticle illuminated by the Gaussian depletion beam at the wavelength of 760 nm was increased (the mean intensity of the scattering image increased from 9.003 to 10.341 for the Gaussian beam and from 6.939 to 7.520 for the donut-shaped beam). Importantly, the distorted donut-shaped wave front recovered the uniform intensity distribution after combining the corrected phase (see Figure S3 of the Supporting Information).

**Figure 7:**
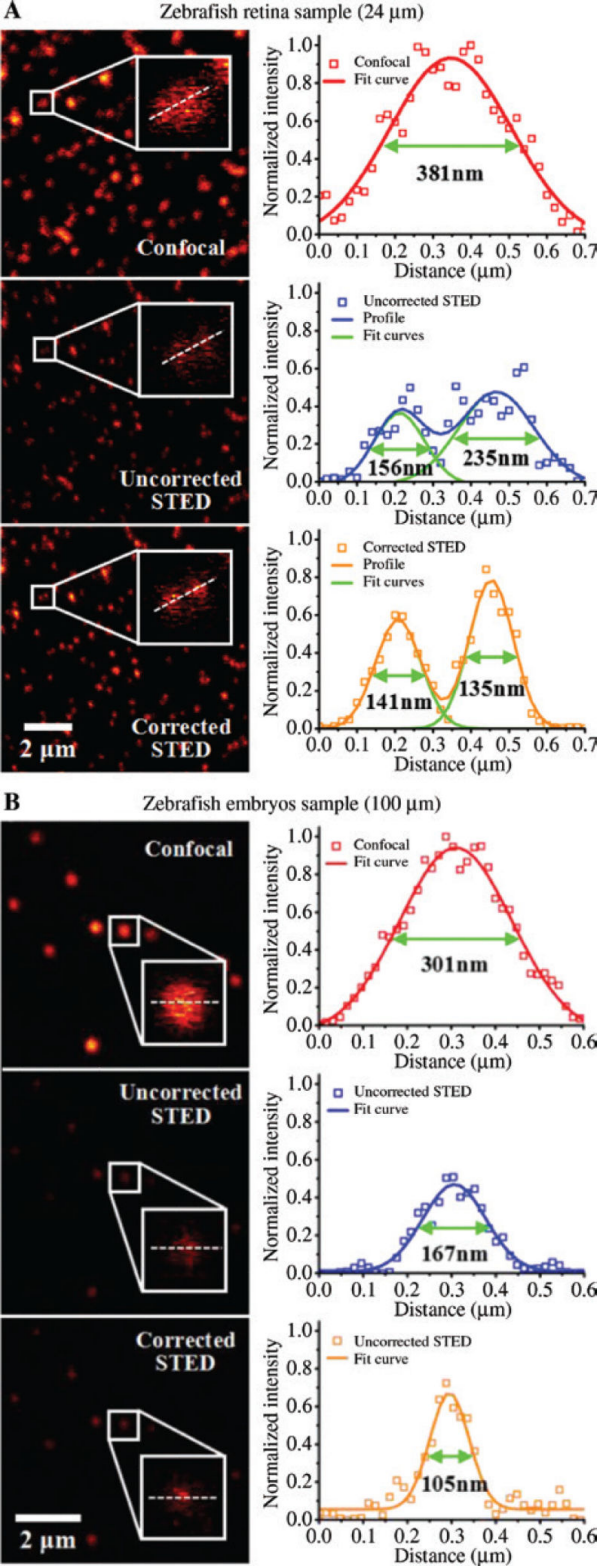
Full aberration correction in zebrafish samples. (A) Images of 100 nm fluorescent microspheres through zebrafish retina with the thickness of 24 μm, and the normalized intensity profiles along white dotted lines. (B) Images of 100 nm fluorescent microspheres though zebrafish embryos section with the thickness of 100 μm, and the normalized intensity profiles along white dotted lines.

Figure 7A shows the images of 100 nm fluorescent microspheres through the zebrafish retina and the normalized intensity profiles along the white dotted lines. The images were obtained at an excitation power of 45 μW. In the uncorrected STED images at the depletion power of 17 mW, the resolution has a slight improvement compared to that in the confocal image, but the signal intensity decreased severely. It illustrated that the wave front of the depletion beam has been distorted through the zebrafish retina with a thickness of 24 μm. However, the situation is different in the corrected STED image. By fitting the intensity profiles along the white dotted lines in the white area of the two STED images with a Gaussian function, two full-widths at half-maximum (FWHMs) can be obtained, which are 156 nm and 235 nm in the uncorrected STED image and 141 nm and 135 nm in the corrected STED image. The FWHM of the PSF provides a useful criterion for the resolution of a microscope, which is often used to determine the imaging resolution in STED microscopy. Therefore, the reduction in FWHM is equivalent to the improvement of imaging resolution. Additionally, the signal intensity was also increased in the corrected STED image compared to that in the uncorrected STED image. More results can be observed from Figure S4 of the Supporting Information, of which the images were obtained across different regions at the depletion power of 15 mW and 20 mW, respectively.

A better image quality can also be achieved in zebrafish embryo samples after performing the full aberration correction. As shown in Figure 7B, the thickness of the zebrafish embryo section was 100 μm and the STED images were obtained at the depletion power of 30 mW. Compared to the uncorrected STED image, the corrected STED image showed a significant improvement in both imaging resolution and signal intensity. It illustrated that the corrected phase can compensate for the distorted wave front to improve the intensity distribution symmetry of the donut-shaped depletion beam when it passed through the zebrafish embryo section. The results showed that the quality of the depletion beam at the focal plane is a crucial factor in performing STED super-resolution imaging. Full aberration correction to the depletion beam is beneficial for the improvement of image quality and the application of STED microscopy in deep tissue imaging. More results about the full aberration correction in different section thicknesses (25 μm and 50 μm) can be seen in Figure S5 of the Supporting Information, of which the images were obtained at the depletion power of 10 mW and 15 mW, respectively. Figure S6 shows the comparison of FWHM before and after the full aberration correction in the two types of zebrafish samples. The reduction in mean FWHM is equivalent to the improvement of resolution. When the thickness of the zebrafish embryo section is larger than 100 μm, the excitation intensity through the sample will become much lower due to aberrations in the excitation path. In this case, the aberrations to the excitation beam must be taken into consideration.

**Figure 8:**
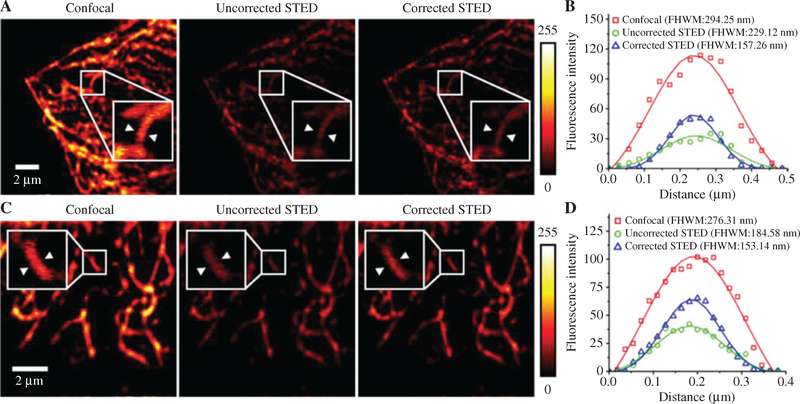
Full aberration correction in Hela cell sample. (A) Images of tubulin structures in a HeLa cell labeled with ATTO647N in the field of view of 15 × 15 μm^2^. (B) Fluorescence intensity profiles marked by white arrows in (A). (C) Images of tubulin structures in a HeLa cell labeled with ATTO647N in the field of view of 10 × 10 μm^2^. (D) Fluorescence intensity profiles marked by white arrows in (C).

Finally, tubulin structures in HeLa cells labeled with ATTO647N were used to demonstrate the capability of GA in full aberration correction of biological structures. In the preparation of the HeLa cell sample, the detection of Anti-α tubulin (Abcam, Cambridge, UK) was performed using secondary antibodies labeled with ATTO647N (Sigma-Aldrich, St. Louis, MI, USA). The detailed steps can be found in the literature [32]. The signal feedback is necessary in the process of aberration correction, but the depletion laser beam cannot excite fluorescence; thus, the back-scattering signal of gold nanoparticles is still necessary. With that, the entire solution in a culture dish was removed after the tubulin structures were dyed, and gold nanoparticles (10 μL) were attached to the sample then mounted in 97% TDE. The gold nanoparticles were dispersed and fixed at different depths (2 ~ 8 μm from the bottom of the culture dish) in the sample. Figure 8 shows the images of tubulin structures in HeLa cells labeled with ATTO647N and fluorescence intensity profiles marked by white arrows. The confocal images were obtained at an excitation power of 45 μW, and the STED images were obtained at a depletion power of 10 mW. The resolution of confocal images cannot go beyond the diffraction limit of this optical system, and the confocal FWHM of single microtubule is 294.25 nm and 276.31 nm marked by white arrows in Figure 8A and C, respectively. In the uncorrected STED images, the resolution has not improved much but the fluorescence intensity decreased a lot. However, the FWHMs of the same single microtubule in STED images improved from 229.12 nm to 157.26 nm and from 184.58 nm to 153.14 nm, respectively. In addition, the fluorescence intensity of the corrected STED images has improved after full aberration correction. The improvement of imaging resolution and signal intensity represents the improvement of image quality.

## 4 Conclusions

In this paper, a wave front compensation approach based on the GA has been proposed. A SLM mounted in the depletion path was controlled by the GA procedure to change the phase level, so that the distorted wave front of the depletion beam can be accurately recovered at the focal plane. By comparing the quality of the STED images before and after the aberration correction in different samples, the approach has been proven to have the capability of correcting the system-induced aberration and the sample-induced aberration. As a result, both the imaging resolution and the signal intensity were improved in STED images, and the imaging depth reached 24 μm and 100 μm in the samples of zebrafish retina and zebrafish embryo sections, respectively. In this work, aberration correction was focused on the depletion beam to improve the quality of the STED super-resolution images. However, the aberrations could also defocus and diffuse the focusing spot of the excitation beam, resulting in the attenuation of the signal intensity. Dividing the liquid crystal display of the SLM into two parts can be a solution. It can not only simultaneously compensate for the distorted wave front of both laser beams but also reduce the expenditure of the experiment system. In conclusion, super-resolution images with an excellent quality will certainly give an impetus to the application of STED microscopy in the future.

## Supplementary Material

Suppl 1
