# Supplementary material for: Aberration correction for improving the image quality in STED microscopy using the genetic algorithm
Source: Nanophotonics. Author manuscript; Available in PMC 2020 Mar 2. (PMC7051000; doi:10.1515/nanoph-2018-0133)
Supplement: Suppl 1 [file NIHMS1032771-supplement-Suppl_1.doc]

**Supporting Information**

**Aberration correction for improving the image quality in STED microscopy using the genetic algorithm**

Luwei Wang^1,a^, Wei Yan^1,a^, Runze Li^2^, Xiaoyu Weng^1^, Jia Zhang^1^, Zhigang Yang^1^, Liwei Liu^1^, Tong Ye^3,＊^ and Junle Qu^1,＊^

^1^Key Laboratory of Optoelectronic Devices and Systems of Ministry of Education and Guangdong Province, College of Optoelectronic Engineering, Shenzhen University, Shenzhen 518060, P. R. China

^2^State Key Laboratory of Transient Optics and Photonics, Xi'an Institute of Optics and Precision Mechanics, Chinese Academy of Sciences, Xi'an 710119, P. R. China

^3^Department of Bioengineering and the COMSET, Clemson University, Clemson, South Carolina 29634, USA

**Corresponding Authors**

^＊^E-mail: (Junle Qu), [jlqu@szu.edu.cn;](mailto:jlqu@szu.edu.cn;) (Tong Ye), [ye7@clemson.edu](mailto:ye7@clemson.edu).

**Author Contributions**

^a^Luwei Wang and Wei Yan contributed equally to this work.


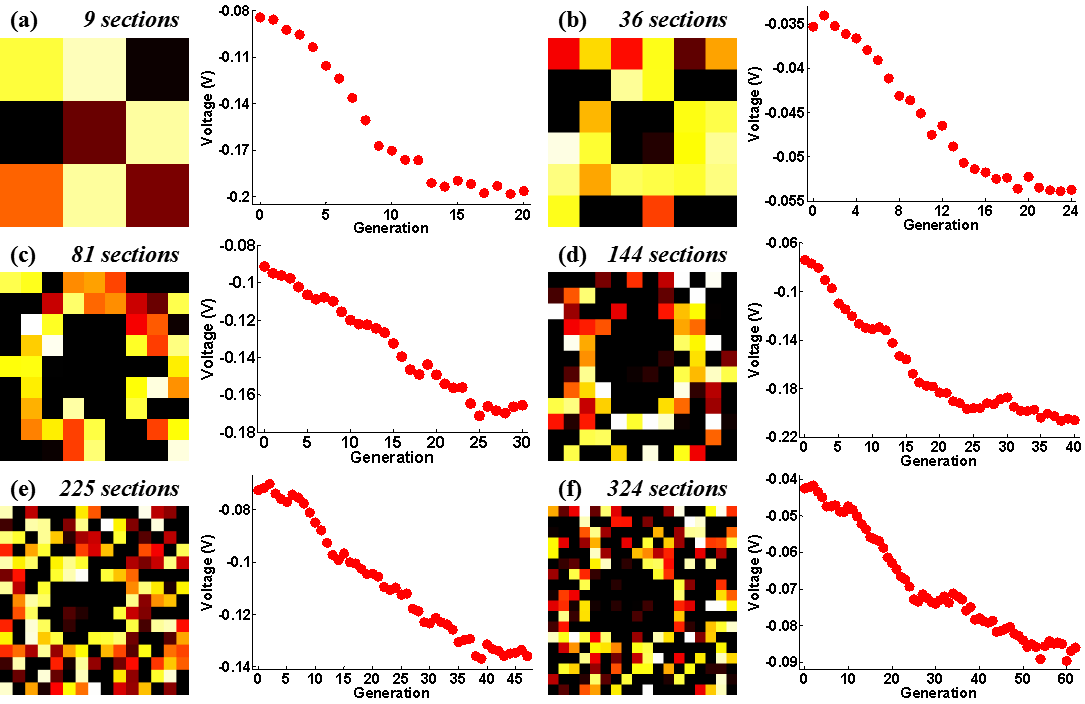


**Figure S1:** The correction of system-induced aberration at different segment number. (a-f) The corrected phases and the voltage changes during the correction in segment number of 9, 36, 81, 144, 225, and 324, respectively.


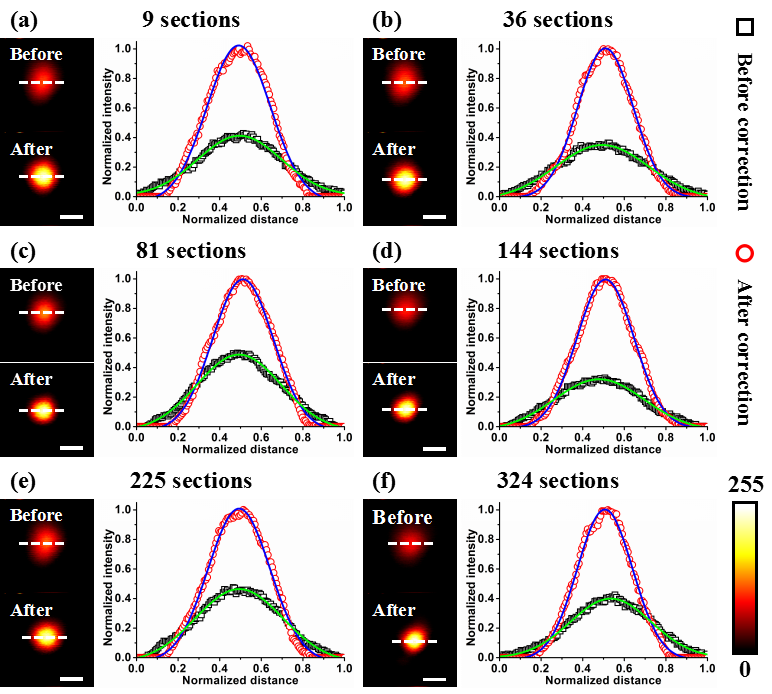


**Figure S2:** The comparison of scattering images of a gold nanoparticle between before and after correcting system-induced aberration in different segment number, and the normalized intensity profiles along white dotted lines. (a) 3×3 segments; (b) 6×6 segments; (c) 9×9 segments; (d) 12×12 segments; (e) 15×15 segments; (f) 18×18 segments. Scale bar: 500 nm.


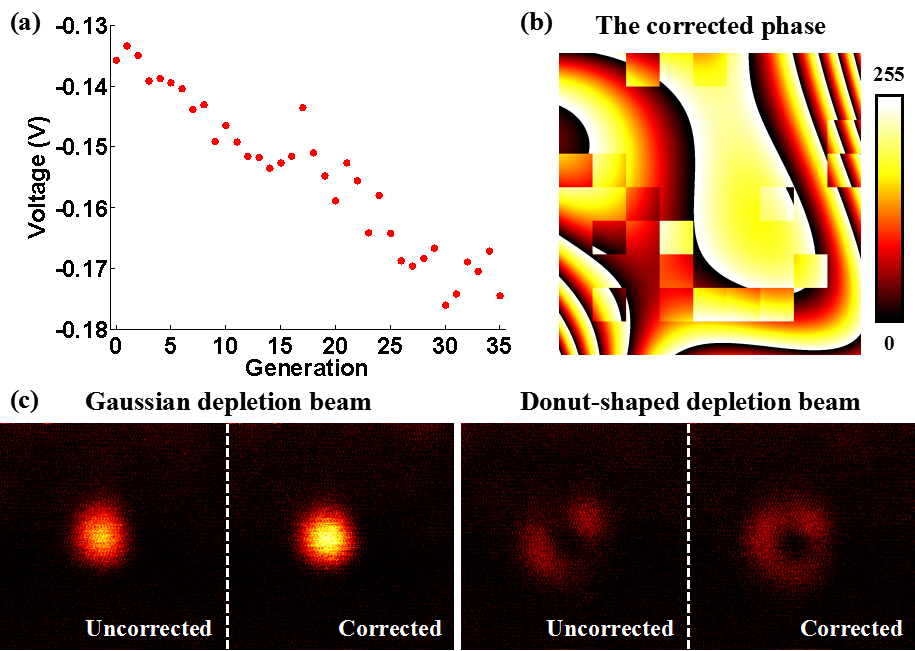


**Figure S3:** The correction of all aberrations in zebrafish retina sample by genetic algorithm. (a) The voltage change during the correction. (b) The corrected phase in the mixed mode of segment and Zernike polynomials. (c) The comparison of scattering images of a gold nanoparticle between before and after all aberration correction illuminated by Gaussian and donut-shaped depletion beam, respectively.


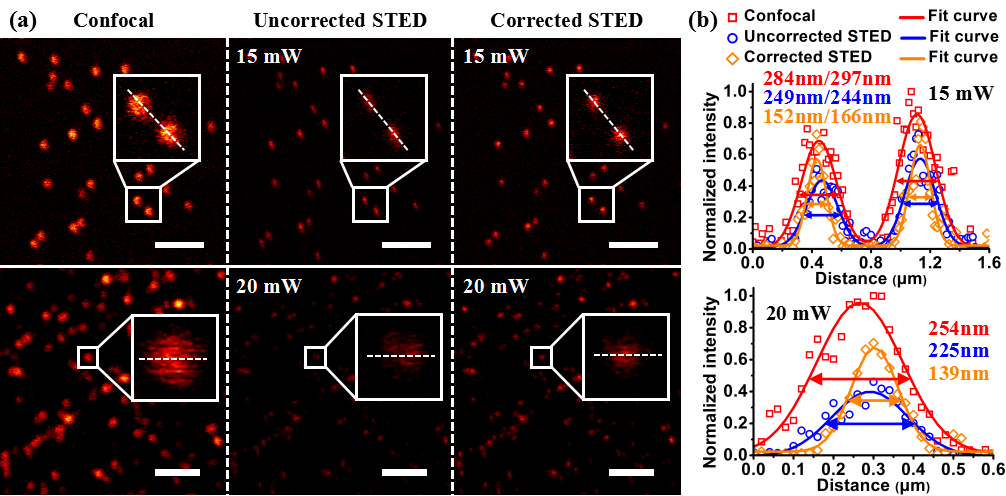


**Figure S4:** (a) Images of 100 nm fluorescent microspheres through zebrafish retina with the thickness of 24 μm, where STED images were respectively obtained at the depletion power of 15 mW and 20 mW. Scale bar: 2 μm. (b) Normalized intensity profiles along white dotted lines in (a). The images of fluorescent microspheres were obtained at different regions. When the depletion power was 15 mW, the two FWHMs (152 nm and 166 nm) in corrected STED image were higher than that in uncorrected STED image (249 nm and 244 nm). When the depletion power was 20 mW, the FWHM of single microsphere (139 nm) in corrected STED image was still higher than that in uncorrected STED image (225 nm). Meanwhile, the signal intensity had an obvious improvement in corrected STED images.


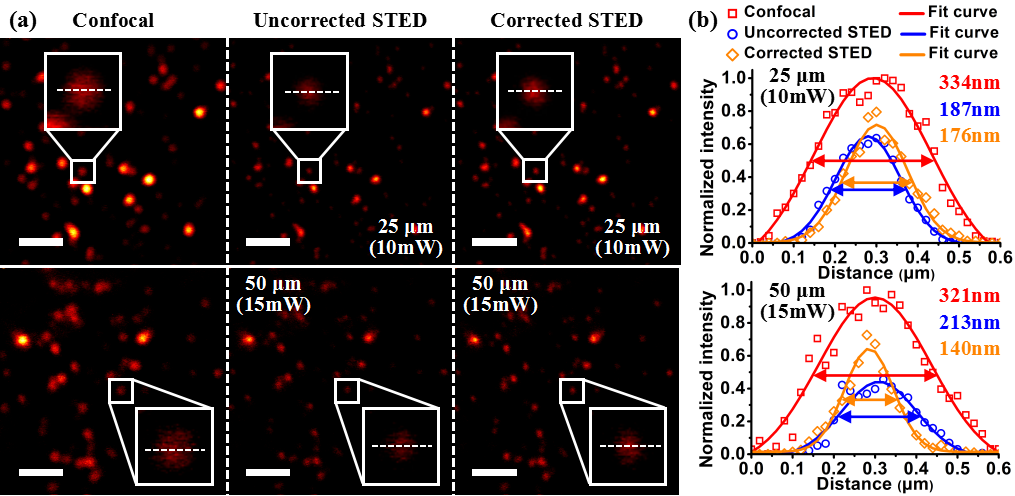


**Figure S5:** (a) Images of 100 nm fluorescent microspheres through zebrafish embryos with the thickness of 25 μm and 50 μm. Scale bar: 2 μm. (b) Normalized intensity profiles along white dotted lines in (a). The images of fluorescent microspheres were obtained at two zebrafish embryos samples at different depletion power. When the depletion power of 10 mW was applied to the sample with thickness of 25 μm, the FWHM (176 nm) in corrected STED image was higher than that in uncorrected STED image (187 nm). When the depletion power of 15 mW was applied to the sample with thickness of 50 μm, the FWHM (140 nm) in corrected STED image was higher than that in uncorrected STED image (213 nm). Accordingly, the signal intensity of two corrected STED images was increased.
